# Confirmation of nasogastric tube placement in critical care

**DOI:** 10.1186/cc12182

**Published:** 2013-03-19

**Authors:** M Moore, R Thomson

**Affiliations:** 1 St Georges Healthcare NHS Trust, London, UK

## Introduction

Placement of nasogastric tubes (NGTs) is commonplace in critical care. Misplacement of NGTs is rare and considered a never event [1]. Strategies to avoid never events (by confirming NGT position) include pH analysis of gastric secretions or chest X-ray confirmation of tube position. For this reason the authors set out to establish the efficiencies surrounding safe placement of NGTs in a 17-bed adult cardiothoracic critical care unit in a large teaching hospital.

## Methods

This small-scale study of 25 NGT placements during a 5-week period collated data supplied by questionnaire by healthcare workers responsible for NGT placements.

## Results

Analysis of Adverse Incident Reports identified no never events of misplaced NGTs within the previous 10 years. This audit revealed that the commonest type of NGT was a radio-opaque tube with stylet (corflo) (92% of placements), with occasional use of the electromagnetic placement system (cortrak) (8% of placements). Sizes 10 (40%) and 12 (56%) were most common. Tube placement was confirmed by: X-ray (72%); pH of aspirates (35%); electromagnetic tube placement (one patient). The time taken from decision to place NGT to use varied (range 15 to 510 minutes). Little distinction was seen in the time taken to use and NGT confirmed by aspirate alone (205 minutes) or by X-ray (220 minutes), although the shortest interval was seen in electromagnetic NGT placement (15 minutes). The cost of NGTs confirmed by aspirate alone was low (approximately £10.00), higher with X-ray confirmation/electromagnetic placement (approximately £45.00).

## Conclusion

Despite the small dataset the results demonstrate a concerning delay in the application of enteral feeding and/or drug administration. Whilst reassuring in the steps taken to avoid never events, this study demonstrates that there may be delays in time-critical administration of enteral medicine or optimal nutritional practices. This study reveals a significant problem with aspirating gastric contents for pH testing, necessitating a large number of X-ray position confirmations. Even if the frequency and volume of gastric aspiration were greater, there is a belief that pH testing may not be sufficiently accurate (since many factors alter patients' gastric pH). It is possible that new technologies such as electromagnetic NGT placement may allow faster/equally safe practices. Further study including cost/benefit analysis will be needed to confirm this.
